# Crossroads of assembling a moss genome: navigating contaminants and horizontal gene transfer in the moss *Physcomitrellopsis africana*

**DOI:** 10.1093/g3journal/jkae104

**Published:** 2024-05-23

**Authors:** Vidya S Vuruputoor, Andrew Starovoitov, Yuqing Cai, Yang Liu, Nasim Rahmatpour, Terry A Hedderson, Nicholas Wilding, Jill L Wegrzyn, Bernard Goffinet

**Affiliations:** Department of Ecology and Evolutionary Biology, University of Connecticut, Storrs, CT 06269, USA; Department of Ecology and Evolutionary Biology, University of Connecticut, Storrs, CT 06269, USA; State Key Laboratory of Agricultural Genomics, BGI-Shenzhen, Shenzhen 518083, China; Key Laboratory of Southern Subtropical Plant Diversity, Fairy Lake 518004, China; State Key Laboratory of Agricultural Genomics, BGI-Shenzhen, Shenzhen 518083, China; Key Laboratory of Southern Subtropical Plant Diversity, Fairy Lake 518004, China; Department of Ecology and Evolutionary Biology, University of Connecticut, Storrs, CT 06269, USA; Department of Biological Sciences, Bolus Herbarium, University of Cape Town, Private Bag, 7701 Rondebosch, South Africa; UMR PVBMT, BP 7151, Université de La Réunion, chemin de l’IRAT, 97410 Saint-Pierre, La Réunion, France; Missouri Botanical Garden, P.O. Box 299, St. Louis, MO 63166-0299, USA; Department of Ecology and Evolutionary Biology, University of Connecticut, Storrs, CT 06269, USA; Institute for Systems Genomics, University of Connecticut, Storrs, CT 06269, USA; Department of Ecology and Evolutionary Biology, University of Connecticut, Storrs, CT 06269, USA

**Keywords:** Funariaceae, reference genome, *Physcomitrium*, bryophyta, HGT, contamination

## Abstract

The first chromosome-scale reference genome of the rare narrow-endemic African moss *Physcomitrellopsis africana* (*P. africana*) is presented here. Assembled from 73 × Oxford Nanopore Technologies (ONT) long reads and 163 × Beijing Genomics Institute (BGI)-seq short reads, the 414 Mb reference comprises 26 chromosomes and 22,925 protein-coding genes [Benchmarking Universal Single-Copy Ortholog (BUSCO) scores: C:94.8% (D:13.9%)]. This genome holds 2 genes that withstood rigorous filtration of microbial contaminants, have no homolog in other land plants, and are thus interpreted as resulting from 2 unique horizontal gene transfers (HGTs) from microbes. Further, *P. africana* shares 176 of the 273 published HGT candidates identified in *Physcomitrium patens* (*P. patens*), but lacks 98 of these, highlighting that perhaps as many as 91 genes were acquired in *P. patens* in the last 40 million years following its divergence from its common ancestor with *P. africana*. These observations suggest rather continuous gene gains via HGT followed by potential losses during the diversification of the Funariaceae. Our findings showcase both dynamic flux in plant HGTs over evolutionarily “short” timescales, alongside enduring impacts of successful integrations, like those still functionally maintained in extant *P. africana*. Furthermore, this study describes the informatic processes employed to distinguish contaminants from candidate HGT events.

## Introduction

Horizontal gene transfer (HGT), also referred to as lateral gene transfer (Zhaxybayeva and Doolittle 2011), is the lateral movement of genetic material between distant branches of the tree of life. This process is ubiquitous among bacteria, facilitating rapid adaptation through exchange of ecologically important genes (Aminov 2011). While HGT is less common in eukaryotes than in prokaryotes, it plays a role in shaping eukaryotic evolution with about 0.04–6.49% of eukaryotic genes originating from HGT from microbes (Van Etten and Bhattacharya 2020). During the evolution of land plants, increasing interactions with rhizosphere microbes, particularly bacteria and mycorrhizal fungi, have enabled occasional horizontal transfer of functional genes between these distantly related lineages (Martin *et al.* 2017). For example, the gene for Killer Protein 4 (KP4) was likely acquired by mosses through HGT from ascomycete fungi (Guan *et al*. 2023). Similarly, the domain of the heterokaryon incompatibility proteins (HET), common in fungal heterokaryon incompatibility genes and involved in self/non-self-recognition, was identified as a truncated form in *P. patens* and may play a role in defenses against microbial pathogens (Sun *et al*. 2020). Van Etten and Bhattacharya (2020) suggested that rather than being merely anecdotal, HGT has been an important evolutionary force driving the adaptations of land plants to new habitats and stressors throughout their evolution, as previously already hypothesized by Yue *et al.* (2012).

Accurately detecting the taxonomic origin of genes and confirming HGT events is challenging. Many reported cases of HGT in published genomes have been proven to be artifacts resulting from contamination that had gone undetected. For example, the initial report that 17% of the genes in the genome of the tardigrade *Hypsibius dujardini* originated from HGT (Boothby *et al.* 2015) has subsequently been revised to only 0.2% (Koutsovoulos *et al.* 2016). Similarly, claims of HGT in mammals also turned out to be erroneous due to bacterial contamination (Douvlataniotis *et al.* 2020), highlighting the need for careful analyses and experimental validation. Systematic screening of 43 arthropod genomes by Francois *et al*. (2020) revealed extensive bacterial contaminants, often outnumbering true HGT events. For example, in the bumblebee *Bombus impatiens*, most contaminating genes were concentrated on just 30 scaffolds. Based on the size and number of contaminant sequences, it was concluded that the genome of the symbiont *Candidatus Schmidhempelia bombi* was co-assembled with that of its host. Strategies to confidently identify HGT, including taxonomic assignment of candidates via BLASTp/DIAMOND, phylogenetic analysis with candidate and donor proteins, synteny analysis of flanking genes, and quantitative PCR validation have been presented to address these issues (Francois *et al.* 2020).

*Physcomitrellopsis africana* (*P. africana*) inhabits transitional zones between grassland and forests in coastal habitats in South Africa. It is currently the sole species of the genus, which likely should also include several species of the paraphyletic genus *Entosthodon* (Wilding 2015). Its genome of 414 Mb, the first genome of an African bryophyte, is presented here. This resource complements those available for *Funaria hygrometrica* and *P. patens* (Rensing *et al.* 2008; Kirbis *et al.* 2022) and thereby constitutes a fundamental resource to further the reconstruction of the evolution of the genome of the model *P. patens* (Rensing *et al.* 2020). Through rigorous contamination screening and verification, 2 genes emerge as candidates resulting from unique HGT events. Informatically validating HGT events in *P. africana* yields evolutionary insights while underscoring the need for stringent standards to support HGT conclusions from genomics data.

## Materials and methods

### Sample collection and culturing

A population of *P. africana* was sampled in October 2010 from a coastal forest in the Eastern Cape Province of South Africa (Dwesa National Park, along trail to chalets behind campground, coordinates 32 °18.222′ S 28 °49.666′, at ± sea level). The voucher specimens collected by Goffinet (collection numbers 10,326 and 10,329, with TAH and NW) are deposited in the George Safford Torrey Herbarium (CONN) under accession numbers CONN00235389 and CONN00235388, respectively. Specimen 10,326 (culture and long read library DNA #5074) provided DNA for long-read sequencing, whereas 10,329 [RNA and Beijing Genomics Institute (BGI)-Seq DNA #5075] provided RNA and DNA for short-read sequencing. Sterile cultures were first established on Knop medium using spores from a single operculate capsule. The gametophytes were harvested, ground, and spread on a rich sandy loam soil in PlantCon tissue culture containers (MP Biomedicals, Solon, OH, USA), and maintained in a growth chamber under 16 h of daylight at about 24°C.

### Genomic DNA and RNA extraction

Gametophytic tissue consisting of stems and leaves of *P. africana* was harvested from fresh soil cultures under a dissecting microscope and ground in liquid nitrogen. DNA was extracted by following a modified protocol by Young (2022). The quality of the DNA sample 5,074 was assessed by quantitative PCR prior to sequencing, yielding a DNA integrity number (DIN) score of 7.0 and concentration of 40.9 ng/μL.

DNA for short-read sequencing was extracted using the NucleoSpin Plant midi DNA extraction kit, following the manufacturer's protocol (Macherey-Nagel, Düren, Germany). DNA quality was evaluated using a Qubit 3.0 Fluorometer (Thermo Fisher Scientific, USA). Total RNA was extracted from approximately 1 g of fresh gametophytic tissue using the RNeasy Plant Mini Kit (Qiagen, Valencia, CA, USA).

### Genome and transcriptome library preparation and sequencing

DNA was prepared for long-read PromethION sequencing through a DNA repair step, generating blunt ends, and ligating sequencing adapters followed by priming of the flow cell, as described in the Oxford Nanopore Technologies Amplicons by Ligation (SQK-LSK109) protocol. The HMW DNA library was sequenced on an Oxford Nanopore PromethION (Center for Genome Innovation, UConn) using a FLO-PRO0002 flow cell. The 2 short-read DNA libraries were prepared following the methods used by Yu *et al* (2020), and were sequenced [150 bp Paired-end (PE)] on 2 lanes of a BGISEQ-500 (BGI-Shenzhen, China).

Approximately 1 μg of RNA was used to generate a paired-end library with an insert fragment size of 200–300 bp of the corresponding cDNA. RNA purification, reverse transcription, library construction, and sequencing were performed at WuXi NextCode (Shanghai, China). The captured coding regions of the transcriptome from total RNA were prepared using the TruSeq RNA Exome Library Preparation Kit. The 2 RNA libraries were sequenced on one lane of an Illumina HiSeq 2,000 (100 bp PE) at the WuXi NextCode (Shanghai, China).

### Quality control of genomic and transcriptomic reads

The genomic short reads were first assessed with FASTQC v.0.11.7 (Andrews 2010). In preparation for assembly with Haslr and Wengan, Sickle v.1.33 with a minimum quality score threshold of 30 (-q) and a minimum length of 50 bp (-l) was employed for read trimming. Nanoplot v.1.21.0 was used to quantify and assess the quality of the nanopore reads. To detect potential contamination, the long reads were aligned against the preindexed bacterial, human, and viral databases with a metagenomic classifier, Centrifuge v.1.0.4-beta (p + h + v; min-hit length was increased to 50 bp) (Kim *et al.* 2016). Reads aligning to the database were removed. The quality of the transcriptomic short reads was assessed with FASTQC v.0.11.7 (Andrews 2010). The reads were trimmed with Sickle v1.33 (Joshi and Fass 2011) with a minimum quality score threshold of 30 (-q) and a minimum length of 40 bp (-l).

### Genome size estimation

A single lane of short-read genomic data from accession 5,075 was employed to estimate the genome size. The k-mer distribution was calculated using Jellyfish v2.2.6 (Marçais and Kingsford 2011) and size estimates were processed with GenomeScope v2.0 (Ranallo-Benavidez *et al*. 2020; Supplementary Fig. 1).

### Transcriptome assembly

The transcriptome was independently assembled to provide protein-level evidence for the structural annotation of the genome, using Trinity v2.6.6 (Grabherr *et al.* 2011), with a minimum contig length of 300 bp. Contigs with minimal read support, post assembly, were removed (fragments per kilobase of transcript per million mapped reads or FPKM > 0.5) with RSEM v1.3.0 (Li and Dewey 2011). Transdecoder v3.0.1 (Haas and Papanicolaou 2012) was used to translate the remaining contigs into open reading frames (ORFs) and remove sequences without a viable frame. To aid Transdecoder in the identification of ORFs, searches against the Pfam database were performed with HMMER v3.1b2 (Zhang and Wood 2003). The Transdecoder annotated the putative transcripts as complete, partial, and internal. Those without a defined start and stop codon (defined as internals) were removed (split_frames.py). The final set of peptide sequences were functionally annotated with EnTAP v0.8.0 (Hart *et al.* 2020) against NCBI's nr protein database and the UniProt/Swiss-prot reference databases. EnTAP was run with contaminant filters that included bacteria, archaea, fungi, and insecta. Transcripts with high confidence alignments to these organisms were removed (contam_removal.py).

### Genome assembly

Hybrid genome assembly, integrating both long- and short-read data, was conducted with MaSuRCA v.4.0.3 (Zimin *et al*. 2013), Wengan v.0.2 (Di Genova *et al*. 2021), and Haslr v.0.8a1 (Haghshenas *et al.* 2020). Additionally, a separate assembly using only long reads as input was conducted with Flye v.2.5 (with three polishing iterations) (Kolmogorov *et al.* 2019).

The Flye, Wengan, and Haslr assemblies were polished following long-read alignment with Medaka v1.3.2 (github.com/nanoporetech/medaka). Post assembly with Wengan, the assembly was filtered to remove scaffolds less than 3 kb. To further improve the accuracy of the assemblies, the hybrid assembly generated by MaSuRCA was polished with the short reads using Pilon v.1.24 (Walker *et al*. 2014). Subsequently, the selected MaSuRCA assembly was processed with Purge Haplotigs v.1.0 (Roach *et al*. 2018).

The Purge Haplotigs pipeline categorized the assembled scaffolds into four coverage levels based on the distribution of mapped reads. This categorization enabled the identification and removal of redundant sequences exhibiting low coverage, presumed to represent erroneous duplicates given the haploid genome. Specific cutoff values of 0, 7, and 65 for the coverage levels were selected to delineate scaffolds to be retained or discarded, based on read depths (Supplementary Fig. 1). The term “allele” is used to describe these redundant sequences for convenience, although it is technically inaccurate for a haploid genome. The coverage analysis and purging allowed isolation of the primary genome sequence from duplications and artifacts generated during assembly.

To evaluate the quality of the assemblies, QUAST v5.2.0 (Gurevich *et al*. 2013) and Benchmarking Universal Single-Copy Ortholog (BUSCO) v4.1.2 (viridiplantae_odb10:425 single copy orthologues) (Manni *et al*. 2021) were employed. Each assembly was also evaluated with Merqury v1.3 (Rhie *et al*. 2020).

### Genome annotation

#### Repeat library construction and masking

The repeat library for the final MaSuRCA assembly was generated using RepeatModeler v.2.01 (Flynn *et al.* 2020) with the long terminal repeat (LTR) discovery pipeline enabled. The genome was then soft-masked with RepeatMasker v.4.0.9-p2 using the consensus repeat library from RepeatModeler (Smit *et al.* 2013).

#### Structural and functional genome annotation

The RNA reads were aligned to the soft-masked MaSuRCA assembly with HISAT2 v.2.1.0 (Kim *et al.* 2019) to provide evidence for protein-coding gene prediction. Two gene prediction analyses were run on the soft-masked assembly using BRAKER v.2.1.5 (Brůna *et al.* 2021), one with RNA-Seq alignment evidence and 1 with protein evidence originating from de novo assembled transcriptome. Gene predictions from both BRAKER runs were integrated with TSEBRA v.1.0.3 (Gabriel *et al.* 2021). From this point, separate assessments were conducted on the RNA-Seq evidence gene predictions (BRAKER) and the final TSEBRA gene predictions to select the best approach. Putative genes were removed from both sets if they did not contain a complete protein domain. This filter was applied with Interproscan v.5.35–74.0 (Jones *et al.* 2014) using the Pfam database v32.0 (Finn *et al*. 2014). It is worth noting that mono-exonic genes can be the result of fragmented annotations and the target metric of 0.2 (mono:multi-exon gene ratio) is often achieved through protein domain filters (Vuruputoor *et al.* 2022). Metrics for the gene predictions were generated with Another Gtf/Gff Analysis Tool (AGAT; Dainat 2024) and BUSCO. After assessment, the filtered BRAKER gene predictions were selected for functional annotation with EnTAP v.0.10.8 (Hart *et al.* 2020). Functional annotation reports from EnTAP (both sequence similarity search and EggNog taxonomy scope classifications) allowed for the identification of nontarget species scaffolds in the assembly (Huerta-Cepas *et al*. 2019).

#### Assembly-level contaminant filtering

Using the functional annotation results from EnTAP, contaminated scaffolds were removed. Scaffolds with a length of 10 kb or less, and with 40% or more of their total genes classified as archaea, bacteria, or fungi, were removed. Additionally, scaffolds greater than or equal to 10 kb with 55% or more genes classified as archaea, bacteria, or fungi were also excluded. The final annotation was then assessed for the annotation rate using EnTAP, the mono:multi ratio using AGAT, and BUSCO completeness.

#### HGT candidate identification

To identify candidate HGTs in *P. africana*, protein sequence similarity searches were conducted with Diamond v2.1.8 (Buchfink *et al.* 2021). The protein sequences of *P. africana* were aligned against “donor” databases, which included sequences from bacteria, fungi, archaea, and metazoa from NCBI's nr database. Additionally, the same proteins were aligned to “recipient” databases containing sequences from Streptophyta, Tracheophyta, Embryophyta, Viridiplantae, and Spermatophyta (Ma *et al*. 2022). Although these categories are not fully exclusive, each database was utilized separately to systematically assess presence across plants at different evolutionary divergence points.

To identify candidate genes representing putative HGT events unique to *P. africana*, the following criteria were utilized: Genes were required to have between one and four significant sequence alignments (*E*-value <1*e*–5) to microbial donor databases, while exhibiting no significant sequence similarity to plant recipient databases. This range of one to four microbial alignments was selected to capture potential HGTs while avoiding ubiquitous domains shared across many microbes. The lack of hits to plant databases was intended to enrich for *P. africana*-specific sequences, rather than those conserved across plants through vertical inheritance. At this stage, any scaffolds containing only bacterial or fungal genes, without any plant-related genes, were removed from the assembly.

To assess the validity of the 2 proposed HGT candidates, the target proteins were independently aligned to the reference genome with Miniprot v0.7. To examine independent transcriptomic support, the RNA reads were aligned to the genome with HISAT2 v2.1.0 and assembled via StringTie2 v2.2.1 (Kovaka *et al*. 2019).

#### Analyzing HGT candidates from *P. patens*

The 273 putative HGTs previously identified in *P. patens* (Yue *et al*. 2012; Ma *et al.* 2022) were independently searched against the *P. africana* and *F. hygrometrica* protein sets using DIAMOND v2.1.8 (Buchfink *et al.* 2021). DIAMOND searches were conducted with an E-value cutoff of 1e-5 and max target sequences set to 1. Hits against *P. africana* and *F. hygrometrica* were collected and merged to generate a summary table with *P. patens* HGTs and the respective top hits in each species.

### Scaffolding the genome to chromosome scale

A single HiC library was prepared for *P. africana* using Proximo Hi-C kit (Phase Genomics) and sequenced as paired-end reads on an Illumina NovaSeq platform (Center for Genome Innovation, UConn). The reads were aligned to the MaSuRCA genome assembly using Burrows-Wheeler Aligner (BWA) v0.7.17 but did not provide sufficient coverage due to contamination. Instead, the *P. patens* T2T reference genome assembly (Zhao 2023; Bi *et al.* 2024) was used to scaffold the *P. africana* assembly to chromosome-scale with RagTag v2.1.0 under default parameters, except a minimum fragment size threshold of 1 kb was specified.

### Comparative genome analyses

A comparative analysis of the protein-coding gene space was conducted with OrthoFinder v.2.5.1 (Emms and Kelly 2019) with *F. hygrometrica* (Kirbis *et al.* 2022) and *P. patens* v3 (Lang *et al.* 2018). To provide a preliminary estimate of gene family size dynamics, gene counts from each species in the assembled orthogroups were categorized as neutral, expanded, or contracted. The first and third quartiles were calculated for the distinct gene counts within a gene family for each species. If the number of genes from a species was lower than the first quartile or higher than the third quartile, then the gene family was categorized as “contracted” or “expanded”, respectively. If the number of genes did not fit with either of these 2 criteria, then the gene family was considered “neutral”. The longest gene for each orthogroup was used to assign functional attributes to all genes in the group from the original EnTAP annotation. If the longest gene did not originate from *P. africana*, then the functional annotation was derived from either *P. patens* or *F. hygrometrica*.

GOSeq (Young *et al.* 2010) enrichment analysis was performed in R v4.2.0. Gene ontology (GO) terms were extracted for each gene from the EnTAP run. Enrichment analysis was investigated separately for the *Biological Process* and *Molecular Function* GO categories. Paralogs of Light-Harvesting Complex (LHC), Serine/Threonine-protein kinase (STN) 7, and STN8 were identified with Diamond v2.8.1 (*E*-value <1*e*–5).

### Whole genome duplication analysis

Chromosome-scale genomes of *F. hygrometrica* and *P. patens* were assessed with the reference genome generated for *P. africana*, with wgd (v.2.0.19) to characterize whole genome duplication (WGD) events (Chen *et al.* 2024). Each species was compared against itself. Nucleotide coding sequences (CDS) were used as input to Blast & Markov clustering (MCL) (Altschul *et al.* 1997; van Dongen 2000). A Ks distribution was constructed using the “ksd” subcommand (Yang 2007; Katoh and Toh 2008; Price *et al*. 2010). Next, a collinearity analysis was performed with “syn” using the structural genome annotations (Proost *et al*. 2011). Model fit was evaluated with the Bayesian and Akaike information criterion (BIC/AIC).

## Results and discussion

### Sequencing and quality control of genomic reads

The single Oxford Nanopore Technologies (ONT) run generated 16 million reads (N50: 7,518 bp; 127× coverage: 404 Mb; 116× coverage: 440 Mb; Supplementary Table 1). Centrifuge filtering reduced this set to approximately 14 M reads (N50: 4,561 bp; 80× coverage: 404 Mb; 73× coverage: 440 Mb). The primary contaminants included bacteria from *Xanthomonadaceae* (10.68%) followed by *Bradyrhizobiaceae* (0.7%). The short-read genomic libraries (100 bp PE) generated 529 M reads. Following trimming with Sickle, 360 M reads remained (178× coverage: 404 Mb; 163× coverage: 440 Mb; Supplementary Table 2). Coverage estimates are provided for both the original estimate (404 Mb) and the final assembled genome size (440 Mb).

### Transcriptome assembly

A total of 36.58 M RNA short reads were generated. Following quality filtering using Trimmomatic, the dataset was reduced to 31.84 M reads. The de novo assembly process with Trinity yielded 143,150 contigs. After expression filtering via RSEM, the number of contigs was reduced to 123,373. Identifying ORFs in the contigs resulted in 110,852 successfully translated transcripts. The average sequence length of these translated ORFs was 803 bp, with an N50 value of 1,038 bp. To enhance the quality of the transcriptome assembly, internal sequences and putative contaminants were removed, resulting in 74,997 total transcripts. After removing internal sequences and contamination, the total unique sequences with a sequence similarity search alignment are 51,982 (69.3%). An assessment of contamination revealed that 8,720 transcripts, 16.78% of the transcriptome, originated from various sources such as amoeba (0.06%), bacteria (0.64%), fungi (15.93%), or insecta (0.12%), as identified by EnTAP. A further 15,500 (18.5%) sequences remained unannotated. The final BUSCO score of the remaining transcripts was C:82%[D:35.7%]. A total of 30,657 (36.6%) transcripts aligned to *P. patens* proteins.

### Genome assembly

#### Initial assembly

**Fig. 1.**
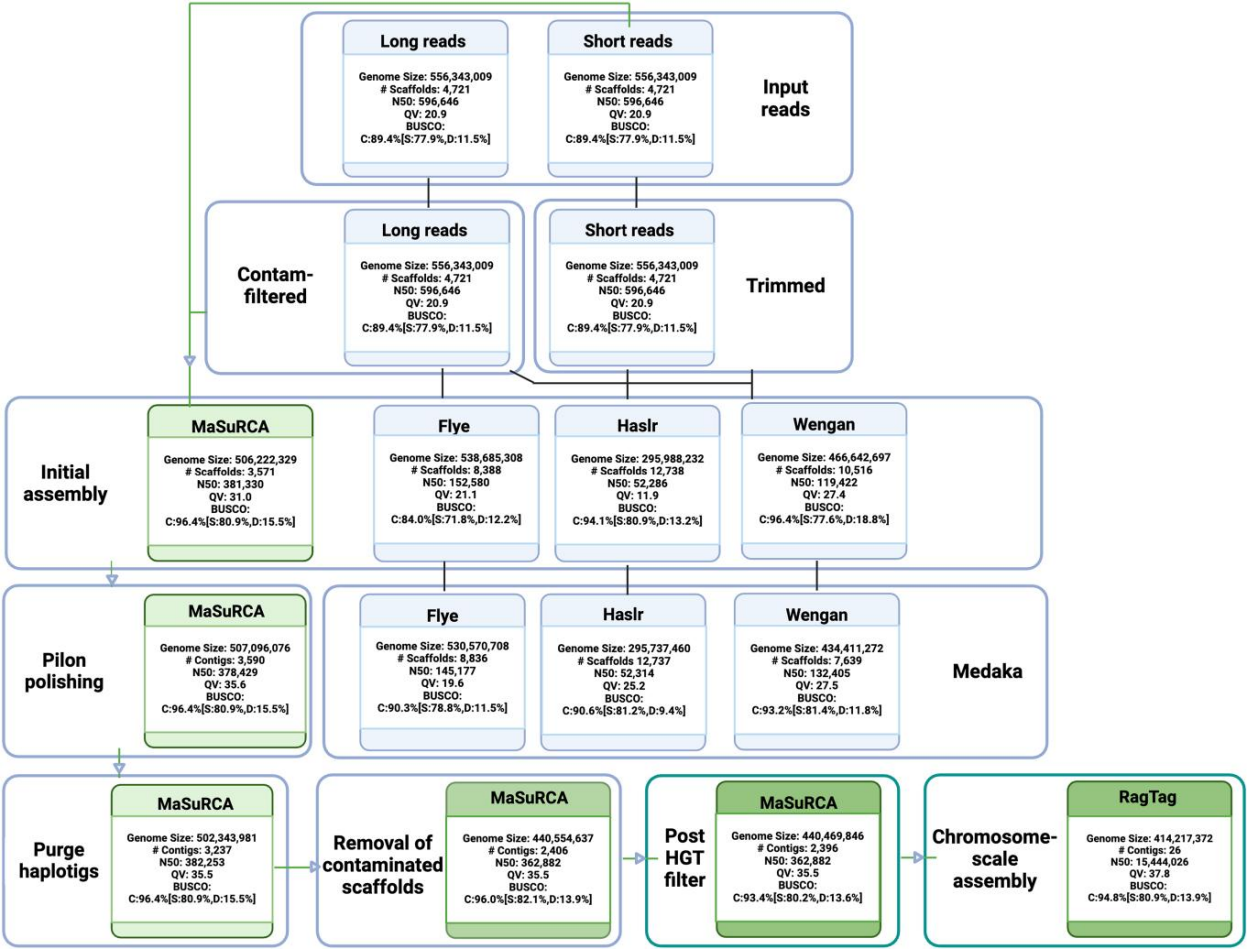
Workflow and statistics for read QC, genome assembly, polishing, and haplotype phasing. Short reads generated from BGI-seq were subject to trimming and the long reads (ONT) were filtered for contaminants with Centrifuge. Filtered and trimmed reads were utilized as input for four different genome assembly software tools: Flye (long-read only), Haslr, Wengan, and MaSuRCA. The MaSuRCA assembly was selected for further analysis. After Pilon polishing, the MaSuRCA assembly was used as input for Purge haplotigs. The final assembly was phased with Purge haplotigs. A total of 831 scaffolds were removed as a result of contaminant filtering using EnTAP following structural genome annotation. Ten additional scaffolds were removed after HGT candidate assessment. The assembly was then scaffolded against the *P. patens* V4 genome using RagTag, capturing 94% of the total assembly across 26 chromosomes. Summary statistics derived from Quast (N50—scaffold/contig length at which 50% of the genome is contained in scaffolds/contigs of this size or greater), BUSCO, and Merqury (QV—Quality Value, an assessment of assembly accuracy) are displayed for each process.

Multiple genome assembly approaches were employed to generate comprehensive draft assemblies of the *P. africana* genome (Fig. 1). The long-read approach, Flye, assembled 538.68 Mb in 8,388 scaffolds, with an N50 of 152.58 Kb. The BUSCO completeness score was C:84.0% [D:12.2%] and the Merqury Quality Value (QV) score was 21.1. Among the hybrid approaches, Haslr produced a 295.98 Mb reference distributed across 12,738 scaffolds, with an N50 of 52.29 Kb. The BUSCO score was C:94.1% [D:13.2%], and the QV score was 11.9. Wengan assembled a total of 466.64 Mb across 10,516 scaffolds, with an N50 of 119.42 Kb, a BUSCO score of C:96.4% [D:18.8%], and a QV score of 27.4. Finally, MaSuRCA assembled 506.22 Mb across 3,571 scaffolds, with an N50 of 381.33 Kb, (BUSCO: C:96.4% [D:15.5%], and a QV score of 31.0).

#### Polishing and improving genome assembly

The Medaka polished Flye assembly had a genome size of 530.57 Mb in 8,386 scaffolds, and the N50 was decreased to 145.17 Kb. The BUSCO dropped to C: 90:3% [D:11.5%]. The QV score decreased to 19.6. Polishing of the Haslr assembly resulted in a genome size of 295.73 Mb, across 12,737 scaffolds. The N50 remained almost unchanged at 52.31 Kb, and the BUSCO score reduced to C:90.6% [D:9.4%]. The QV score more than doubled to 25.2. Filtering for scaffolds <3 Kb, followed by Medaka, resulted in a smaller genome size for the Wengan assembly at 434.41 Mb across 7,639 scaffolds. The N50 increased to 132.40 Kb. The BUSCO score was reduced to C:93.2% [D:11.8%], and the QV increased slightly to 27.5.

Polishing with Pilon had very minimal influence on the completeness, as expected, and substantial impact on the accuracy of the MaSuRCA assembly. The polished MaSuRCA hybrid assembly size increased slightly to 507.10 Mb across 3,590 scaffolds, with a slightly decreased N50 of 378.43 Kb. The BUSCO completeness remained the same at C:96.4% [D:15.5%], and the QV score increased to 35.6.

#### Refinement of genome assemblies with purge haplotigs

The MaSuRCA assembly was selected for further refinement. This decision was based on the overall quality assessed by Merqury, BUSCO completeness, and overall contiguity. The MaSuRCA assembly was refined with purge haplotigs, which reduced the assembly length to 502.34 Mb across 3,237 scaffolds with an N50 value of 382.25 Kb. The BUSCO completeness score remained the same (e.g. 96.4%) and the QV score was minimally reduced to 35.5. At 502.34 Mb, the assembled genome is ∼100 Mb longer than the k-mer based estimate (440 Mb, Supplementary Fig. 2).

### Genome annotation

#### Repeat identification

RepeatModeler produced a library containing 580 unique repeats that were used to softmask 50.22% of the final assembly (Supplementary Table 3). Of the repeats, 36.53% were composed of *Ty3/Gypsy* and 1.46% of *Ty1/Copia*. This is similar to the pattern in *P. patens*, wherein approximately 57% of the genome is composed of repeat elements, with LTRs, particularly the *Gypsy* family, accounting for 48% of the masked genome (Lang *et al.* 2018). These findings align with observations by Kirbis *et al.* (2022), suggesting a common pattern regarding the activation of *Gypsy* elements in *P. africana* and *P. patens*. By contrast, *F. hygrometrica*, which diverged from *P. patens* 60–80 MYA (Medina *et al.* 2018; Bechteler *et al.* 2023), exhibits a lower overall repeat estimate of 35%. Here, *Gypsy* elements contribute less to the LTR content, i.e. roughly 10%, whereas *Copia* elements contribute 17%.

#### Protein-coding gene identification

The *P. africana* RNA-Seq library had an overall alignment rate of 78.43%, likely due to contaminant content in the source extraction. Regardless, a substantial set of 37 M reads were retained, exceeding the minimum needed for prediction. The first BRAKER2 predictions, using RNA-Seq evidence alone, generated 60,917 protein-coding genes with a BUSCO completeness score of C:95.8% [D:15.3]. The mono:multi exonic ratio was 0.52. With only protein evidence, BRAKER2 generated 37,752 gene predictions with a BUSCO score of C:46.1% [D:16.0]. Merging these predictions, as recommended by TSEBRA, resulted in a set of 45,737 genes with a BUSCO score of C:91.3% [D:15.1], and a mono:multi exonic ratio of 1.06. The gene prediction set generated with RNA-Seq alignment evidence exclusively was selected for further refinement due to its higher BUSCO score and lower mono:multi ratio compared with the merged TSEBRA transcripts. Through protein domain filtering (InterProScan filter), the number of mono-exonic genes was further reduced from 20,081 to 12,696, producing a total of 23,561 genes (BUSCO: C:88.5% [D:13.8%]; mono:multi ratio: 0.08).

Based on the functional annotations of the gene space, we removed 831 scaffolds and 1,250 genes related to contaminants. This also reduced the assembly length to 440 Mb in 2,406 scaffolds with an N50 of 363 Kb, and an assembly BUSCO score of C:96% [D:13.9%]. This substantial reduction led to an assembly within ∼40 Mb of the original k-mer-based estimate (e.g. 404 Mb).

The annotated protein-coding gene space in the contaminant-filtered assembly included 1,708 mono-exonic and 21,853 multiexonic genes. A BUSCO score of C:93.8% [D:13.6%] and mono:multi ratio of 0.08 was reported. Reciprocal BLAST conducted by EnTAP produced an annotation rate of 78%, of which 93.6% aligned to *P. patens* (Table 1; Supplementary File 1). This contrasts with the annotation rate of 50% in *F. hygrometrica* (Kirbis *et al*. 2022), attributed to significant divergence time from *P. patens*, and the lack of closely related species in public genomic databases (Kirbis *et al.* 2020; Rahmatpour *et al.* 2021). The higher annotation rate in this study suggests that many genes found in *P. patens,* but not *F. hygrometrica*, may have been acquired in the ancestor of the *Physcomitrellopsis*–*Entosthodon*–*Physcomitrium* clade (Medina *et al.* 2019).

**Table 1.** Genome annotation statistics for *P. africana*.

Annotation method	Total genes	BUSCO (viridiplantae)	Mono: Multi ratio	Annotation rate
BRAKER (RNA)	60,917	C:95.8% [D:15.3]	0.52	65%
BRAKER (protein)	37,752	C:46.1% [D:16.0]	0.95	73%
TSEBRA (BRAKER RNA + BRAKER protein)	45,737	C:91.3% [D:15.1]	1.02	74%
BRAKER (RNA) + InterProScan filter + scaffold contam filter)	23,561	C:93.8% [D:13.6%]	0.08	78%
BRAKER (RNA) + InterProScan filter + scaffold contam filter + HGT filter)	23,535	C:88.5% [D:11.3%]	0.07	83%
Scaffolded to 26 chromosomes with RagTag	22,925	C:86.4% [D:11.1%]	0.07	75%

### Scaffolding the genome assembly to chromosome-scale

The T2T assembly of *P. patens* (V4; Zhao 2023; Bi *et al*. 2024) was used to scaffold the *P. africana* genome assembly. The final assembly contained 440.65 Mb in 583 scaffolds with an N50 length of 15.04 Mb. The 26 largest pseudomolecules, corresponding to the *P. patens* V4 haploid chromosome number, covered 414.21 Mb or 94% of the 440mMb assembly. From here on, we will refer to these pseudomolecules as chromosomes, numbered according to their lengths. The final genome assembly had a BUSCO completeness of C:94.8% [S:80.9%, D:13.9%], F:1.2%, M:4.0%, n:425, and a total number of 22,925 protein coding genes. While most of the annotated gene space was retained, the BUSCO duplication score remained at 13%, compared with 11–15% for the *F. hygrometrica* and *P. patens* genomes (Kirbis *et al.* 2022). Of the 557 contigs/scaffolds that did not contribute to the 26 chromosomes, 185 (33.34%) contained at least 1 protein coding gene annotation from the first annotation. Among those, 3 (1.6%) contained at least 1 gene that was annotated as a contaminant but did not meet the original thresholds that removed full scaffolds.

### Comparative genome analysis

**Fig. 2.**
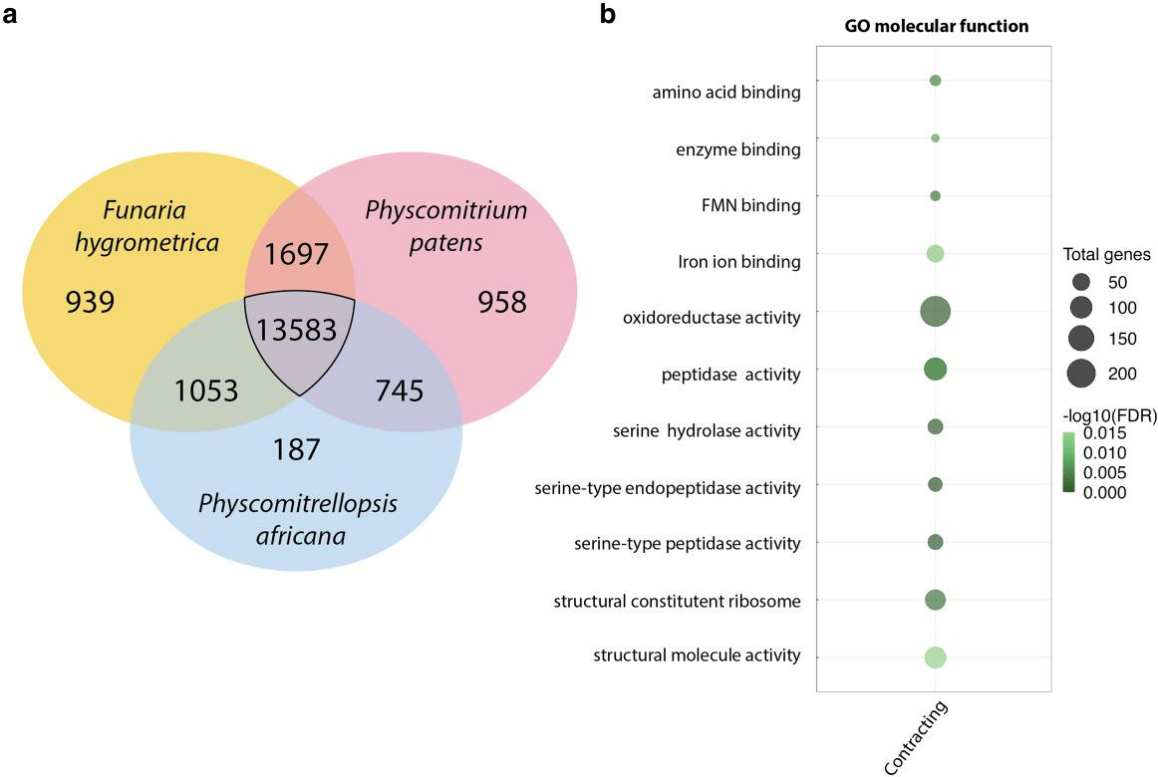
a) The total shared and unique orthogroups among *F. hygrometrica*, *P. africana*, and *P. patens*. b) Enriched *Molecular function* GO terms for gene families in a comparative analysis between *P. africana*, *P. patens*, and *F. hygrometrica*, showed contraction in the GO terms related to oxidoreductase activity, as well as serine peptidase activity and FMN binding for *P. africana*. The size of each bubble represents the number of gene families, and the gradient indicates the significance level of enrichment—darker shades denote more significant enrichment.

**Fig. 3.**
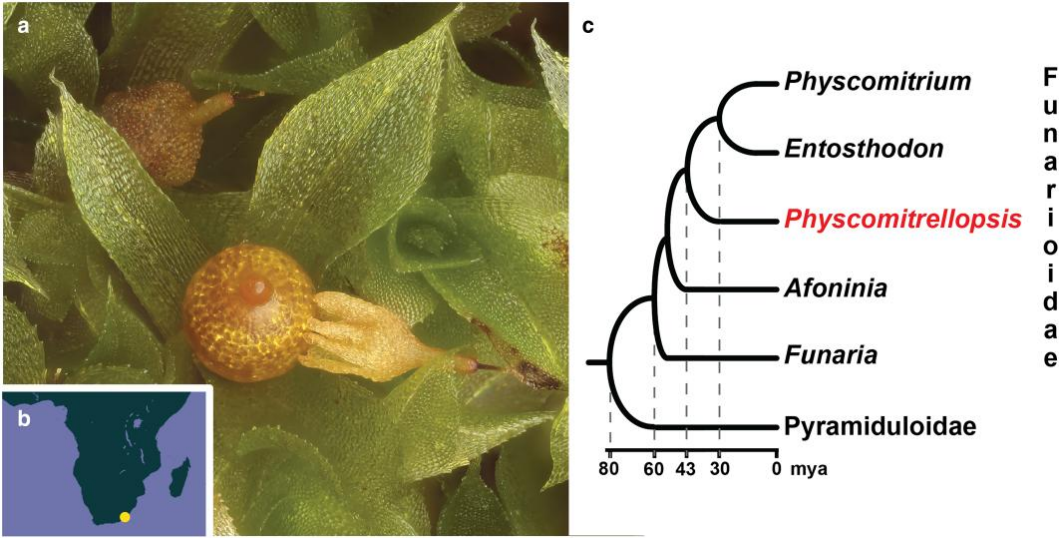
a) *P. africana* exhibits a reduced architectural complexity of the sporophyte similar to that observed in *P. patens*, namely, a sessile, aperistomate, and cleistocarpous sporangial capsule. b) The known geographic distribution of *P. africana*, a rare narrow species endemic to the Eastern Cape Region in South Africa. c) Phylogenetic relationships and chronology of the evolution of *Physcomitrellopsis* based on Medina *et al*. (2018).

The comparison of the genomes of *P. africana*, *F. hygrometrica*, and *P. patens* reveals that they share 13.5 K orthogroups, but also hold 187, 939, and 958 unique orthogroups, respectively. *F. hygrometrica* and *P. africana* exclusively share more (1,053) orthogroups than *P. africana* and *P. patens* (745) (Fig. 2a), despite the latter sharing a more recent unique common ancestor (Fig. 3). Thus, whereas Funariaceae share a rather conserved architecture of their vegetative body even after at least 60 MY of divergence, their gene space varies considerably (Kirbis *et al.* 2022). Such differentiation was previously noted based on highly diverging transcriptomes (Rahmatpour *et al.* 2021), and interpreted as reflective of strong ecophysiological adaptations, which may be the driving force in bryophyte evolution (Glime 1990). Signatures of 2 ancient WGD events were found in both *P. patens* and *F. hygrometrica*. Previous studies have shown that genes related to regulation were preferentially retained after the first WGD in *P. patens* (Lang *et al.* 2018). Our analysis of *P. africana* also retrieved 2 peaks, in agreement with the 2 WGD events in *F. hygrometrica* and *P. patens*. The Ks peaks were at 0.56 and 0.92, and the AIC and BIC values accorded with 2 WGD events. To assess the gene expansion and contraction analysis, of the putative gene families from the *P. africana* annotation, 809 and 1,694 were, respectively, categorized as expanded and contracted (Supplementary File 2). Enrichment analysis examined through GO's *Biological Process* category revealed 167 expanded terms and 6 contracted terms. By contrast, within the *Molecular Function* GO category, 62 were contracted, and no terms were significantly expanded.

Although expanded *Biological Process* GO terms were excessively broad, orthogroup analysis revealed a pattern of contraction in GO terms pertaining to oxidoreductase activity, flavin mono nucleotide (FMN) binding, and serine protease activity (Fig. 2b). Whether this unique suite of downregulated categories diagnoses photosynthetic properties of *P. africana* only or of the expanded genus sensu Wilding (2015) remains to be tested. The observed changes in GO term enrichment related to oxidoreductase activity, FMN binding, and serine protease activity suggest potential differences in photosynthetic processes between *P. africana* and other moss species.

The complement of LHC genes is expanded in *P. patens* compared with algae and vascular plants (Alboresi *et al.* 2008; Iwai *et al.* 2018). LHC proteins bind chlorophylls and carotenoids to facilitate light absorption and energy transfer to the reaction centers of Photosystems (PS) I and II. The LHC genes are classified into 2 groups: Lhca encodes antenna proteins for PSI (LHCI) whereas Lhcb encodes antenna proteins for PSII (LHCII). Although ancestral land plants contain several LHC homologs, further expansion occurred in *P. patens* after WGD events (Alboresi *et al.* 2008; Rensing *et al.* 2008; Zimmer *et al*. 2013; Sun *et al.* 2023). This led to a larger repertoire of LHC genes compared with the alga *Chlamydomonas reinhardtii* and the vascular plant *Arabidopsis thaliana*. Specific Lhca and Lhcb paralogs are present in multiple copies in the *P. patens* genome compared with 1 to 2 copies in *C. reinhardtii* and *A. thaliana*. The major antenna proteins encoded by Lhcbm also show greater redundancy and diversity in *P. patens* (Iwai *et al*. 2018; Sun *et al*. 2023).

Comparing the genomes of *P. africana, F. hygrometrica,* and *P. patens* reveals both conservation and divergence of LHC genes. For example, *P. africana* has 8 distinct Lhca genes, whereas *P. patens* has 12, and *F. hygrometrica* has 7. Similarly, whereas *P. africana* has seven distinct Lhcb genes, *P. patens* has 12, and *F. hygrometrica* has nine. Finally, *P. africana* has 2 distinct Lhcbm genes, *P. patens* has 14, and *F. hygrometrica* has 8. However, echoing Kirbis *et al*. (2022), more LHC gene duplicates were retained in *P. patens* than in *F. hygrometrica* and *P. africana* (Supplementary Fig. 3, Kirbis *et al*. 2022). Whereas *P. patens* retained multiple LHC paralogs of possible WGD origin or gene duplications, *F. hygrometrica* and *P. africana* may have lost some of this gene redundancy. This pattern of differential retention is further supported by assessing the number of paralogs and orthologues for each LHC gene family across the three genomes (Supplementary Table 4). Although some copies of LHC genes were lost in *F. hygrometrica* and *P. africana*, key STN7 and STN8 kinases involved in photosynthetic acclimation are conserved and retained in all 3 genomes, suggesting retention of core light signaling components.

### Contaminant filtering and the identification of HGT events

**Fig. 4.**
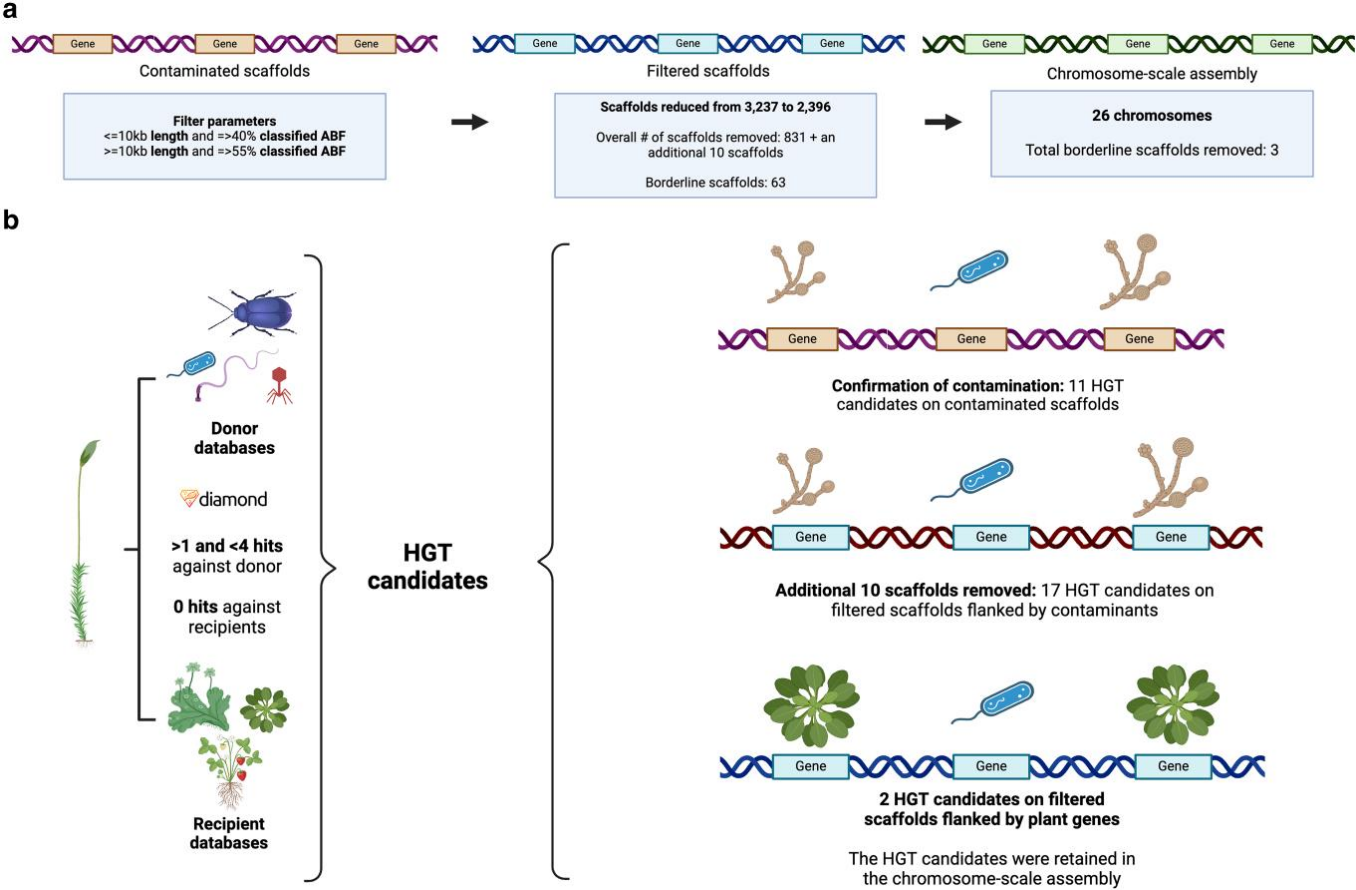
a) Contamination vs HGT in the *P. africana* genome. Parameters used for removing a set of contaminated scaffolds from the draft genome based on functional characterization of the annotated gene space. Scaffolds with a length of 10 kb or less, and those with 40% or more of their total genes classified as archaea, bacteria, or fungi, were removed. Additionally, scaffolds with a length greater than or equal to 10 kb and having 55% or more genes classified as archaea, bacteria, or fungi were also excluded. In total, 831 scaffolds were removed because of this filtering process. Altogether 63 scaffolds contained at least one gene annotated as a contaminant but did not meet the original thresholds for removing full scaffolds. When the chromosome-scale assembly was generated with RagTag scaffolding, 3 of these scaffolds were not placed in the 26 chromosomes. b) Identification of HGT candidates via sequence similarity comparisons of *P. africana* proteins against both “donor” databases (archaea, bacteria, fungal, and metazoan) and “recipient” databases (Streptophyta, Tracheophyta, and Spermatophyta). Proteins with >1 and <4 hits against all donor databases, and no hit against recipient databases, were labeled as HGT candidates. This analysis was conducted on the contaminated scaffolds removed in A, confirming their contamination status. On the post-filtered scaffolds, there were some putative HGTs that were flanked by contaminants. These scaffolds were also removed from the assembly (additional 10), resulting in the retention of 2 HGT candidates in the final analysis.

In plants, horizontal transfer of genes can occur at all phylogenetic depths, via introgression following hybridization between plant species (Pfennig 2021) to acquisition of genetic material from other domains (i.e. bacteria) or phyla of eukaryotes (e.g. fungi) (Soucy *et al*. 2015; Husnik and McCutcheon 2018). Identifying such transfers, and hence the donors, is challenged by the occurrence of the plant's microbiome (Koutsovoulos *et al*. 2016; Francois *et al*. 2020) and hence requires critical screening of sequencing outputs. Filtering before and after the assembly of *P. africana* was employed, illustrating that iterative approaches at all stages enhanced the final quality of the reference. Here, metagenomic tools (Centrifuge; Kim *et al*. 2016), optimized for long reads, identified contaminants before assembly. Since this initial filter did not include the short reads or assess fungal contributions in either sequence set, further filtering was conducted after assembly and annotation. An estimated 22% of the genes were of fungal origin and 31% were of bacterial origin. Scaffolds that contained numerous genes originating from algae, bacteria, or fungi were removed. Separately, the annotated gene space was aligned to a set of “donor” and “recipient” databases. This identified 31 potential HGT events. Following the set of best practices outlined by Francois *et al*. (2020), an additional set of 10 scaffolds was found to be contaminated as the flanking genes of the candidates were not of plant origin. This resulted in their removal (HGT filter). Two unique HGT candidates survived these filters, and the final scaffolding (Fig. 4).

The first HGT candidate, *Pa1_19801.1*, aligns to C-type lectin (CTL) mannose-binding isoform-like XP_032077963.1. C-type lectins are the most frequent binding sites across all plants and animals. In vertebrates, CTLs have a function of pathogen recognition. HGTs in the context of plant defense and immunity are not new and have been identified before (Yue *et al.* 2012; Ma *et al.* 2022). In fact, Ma *et al.* (2022) identified a lectin that was acquired from bacteria. The second candidate, *Pa1_12964.1*, aligns to a hypothetical protein found in *Endogone* sp. FLAS-F59071 (RUS16308.1). While there is no information on the function of this gene, transfers from fungi are not surprising. As mentioned previously, fungal killer proteins KP4 have been acquired by mosses and play an important role in protonemal development (Guan *et al*. 2023).

**Fig. 5.**
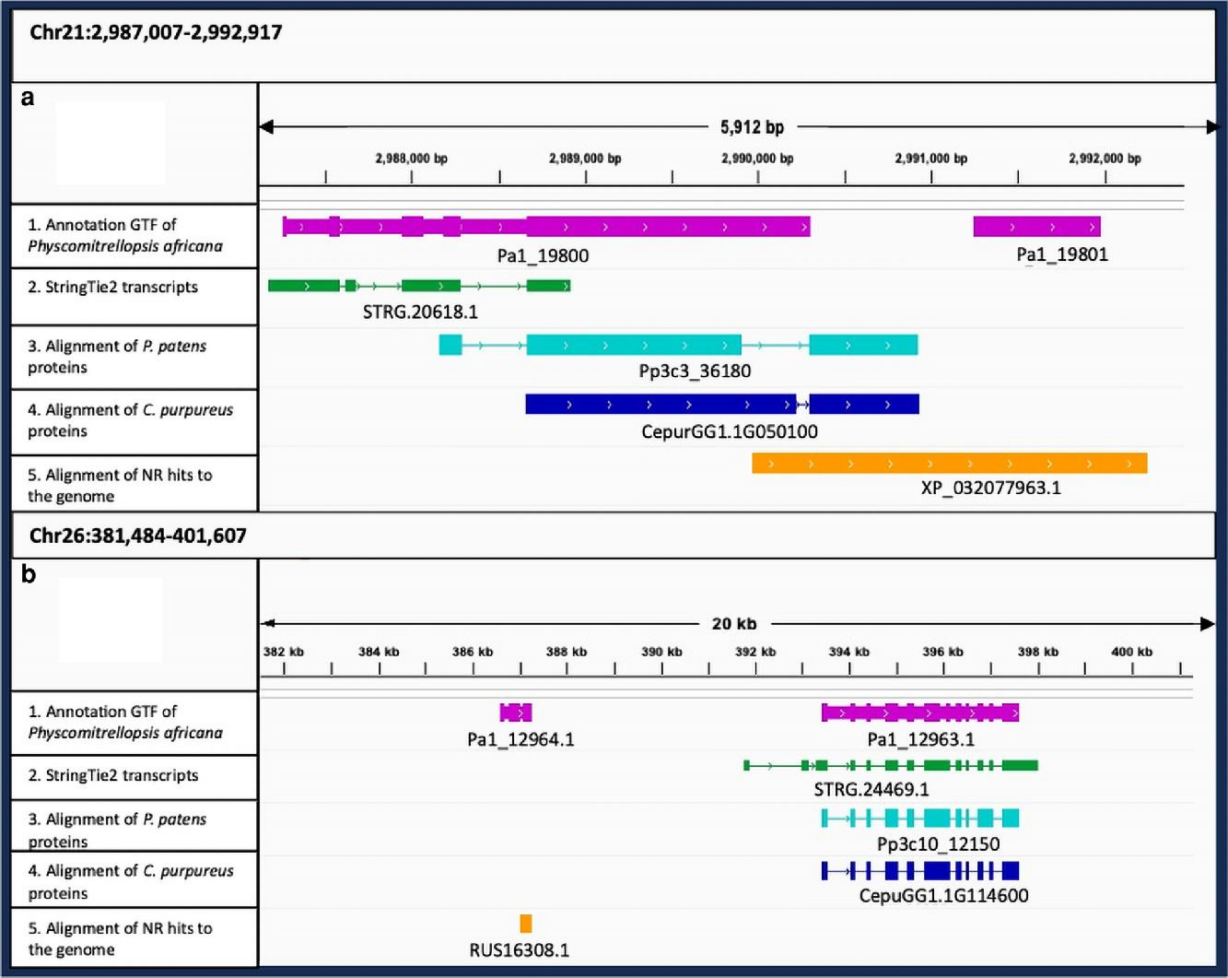
Integrated Genome Viewer (IGV) screens depicting 5 tracks of the *Physcomitrellopsis africana* genome. Track 1 shows the protein-coding structural annotation in context to the genome. Track 2 displays genome-guided transcript assemblies via StringTie2. Tracks 3 and 4 show the alignments of *P. patens* and *C. purpureus* proteins onto Chromosomes 2 and 26. Track 5 illustrates the alignment of the HGT candidate (nr database) to the genome. a) and b) show the HGT candidates *Pa1_19801.1* and *Pa1_12964.1* where the HGT candidate alignment validates the presence of a HGT protein. In both cases, independent RNA assemblies, via StringTie2, were not able to generate a supporting model. None of the other moss proteins from *P. patens* and *C. purpureus* aligned to the region hosting the HGT candidates in *P. africana.*

These 2 candidates were manually validated by comparing alignments of de novo assembled transcriptomes. Both candidates are directly flanked by well-annotated plant (moss) genes. Additionally, aligning the full set of *P. patens* and *Ceratodon purpureus* proteins to the *P. africana* scaffolds produced no alignments in proximity to the candidates, further suggesting that these HGT candidates are specific to *P. africana* (Fig. 5).

#### Analyzing HGT candidates from *P. patens*

The genomes of *P. africana* and *F. hygrometrica* were screened for the 273 putative HGTs previously identified in *P. patens* (Yue *et al*. 2012; Ma *et al*. 2022). Approximately 91 of these genes (33%) were not found in the other 2 species, whereas 15 (5%) were shared only by *P. africana* and *P. patens*, and seven (3%), only by *P. patens* and *F. hygrometrica* (Supplementary Fig. 4). The greater number of shared HGTs between *P. patens* and *P. africana* likely reflects their more recent divergence (at least 40 MYA) compared with that of *P. patens* and *F. hygrometrica* (at least 60 MYA) (Medina *et al*. 2018; Bechteler *et al*. 2023). These findings suggest that HGT may have played a role in the evolution of mosses, potentially facilitating their adaptation to diverse environments over their long evolutionary history dating back 500 million years (Bechteler *et al*. 2023). The life cycle of mosses, with stages where cells are exposed to microbes, such as the egg or zygote within the open archegonium or motile sperm cells, could provide opportunities for the uptake of foreign genetic material from bacteria or fungi (Yue *et al*. 2012; Huang 2013). These vulnerable stages raise the hypothesis that putative microbial symbionts or associates of mosses may have been sources of HTGs, potentially aiding mosses in adapting to harsh environmental conditions encountered as some of the earliest terrestrial plants.

## Supplementary Material

jkae104_Supplementary_Data

## Data Availability

All scripts and data are described in https://doi.org/10.5281/zenodo.11094703. NCBI BioProject ID PRJNA1020579 contains the genomic short reads and nanopore long reads (SRR26596311, SRR26596310, and SRR26666216) and the RNA reads (SRR26586950), de novo transcriptome assembly (GKQB00000000.1), as well as the whole genome assembly (GCA_036785485.1). The annotation files are available at https://doi.org/10.6084/m9.figshare.25724079. Supplemental material is available at G3 online.
